# MANAGEMENT OF ENDOCRINE DISEASE: Dysnatraemia in COVID-19: prevalence, prognostic impact, pathophysiology, and management

**DOI:** 10.1530/EJE-21-0281

**Published:** 2021-08-06

**Authors:** Ploutarchos Tzoulis, Ashley B Grossman, Stephanie E Baldeweg, Pierre Bouloux, Gregory Kaltsas

**Affiliations:** 1 Division of Medicine, Department of Metabolism and Experimental Therapeutics, University College London, London, UK; 2 Department of Endocrinology, OCDEM, University of Oxford, Oxford, UK; 3 Neuroendocrine Tumour Unit, Royal Free Hospital, London, UK; 4 Centre for Endocrinology, Barts and the London School of Medicine, Queen Mary University of London, London, UK; 5 Department of Diabetes and Endocrinology, University College London Hospital NHS Foundation Trust, London, UK; 6 Division of Medicine, University College London, London, UK; 7 Centre for Neuroendocrinology, Royal Free Campus, University College London, London, UK; 8 First Department of Propaedeutic and Internal Medicine, Laiko University Hospital, National and Kapodistrian University of Athens, Athens, Greece

## Abstract

This review examines the prevalence, aetiology, pathophysiology, prognostic value, and investigation of dysnatraemia in hospitalised COVID-19 patients, taking into account all relevant studies published in PubMed and Cochrane Library studies until March 2021. Hyponatraemia is commonly observed in patients with bacterial pneumonia and is an independent predictor for excess mortality and morbidity. However, it remains unknown whether this association applies to coronavirus disease-2019 (COVID-19). Several studies reported a 20–35% prevalence for hyponatraemia and 2–5% for hypernatraemia in patients admitted with COVID-19. In addition, hyponatraemia on admission was a risk factor for progression to severe disease, being associated with an increased likelihood for the need for invasive mechanical ventilation, with an odds ratio (OR) of 1.83–3.30. Hyponatraemia seems to be an independent risk factor for mortality, with an OR of 1.40–1.50 compared to normonatraemia, while hypernatraemia is related to even worse outcomes than hyponatraemia. Furthermore, preliminary data show an inverse association between serum sodium and interleukin-6 levels, suggesting that hyponatraemia might be used as a surrogate marker for the risk of a cytokine storm and the need for treatment with interleukin antagonists. In conclusion, dysnatraemia is common and carries a poor prognosis in COVID-19 patients, indicating that it may play a future role in risk stratification and individualising therapy.

## Introduction

Numerous studies have demonstrated a U-shaped relationship between serum sodium concentration and in-patient mortality in general hospital populations, with both hyponatraemia and hypernatraemia (defined as serum sodium levels below 135 mmol/L and above 145 mmol/L, respectively) being independent risk factors for mortality (1, 2). Many studies in patients with community-acquired pneumonia (CAP) have reported that hyponatraemia, mainly attributable to the syndrome of inappropriate antidiuretic hormone secretion (SIADH) or hypovolaemic hyponatraemia (3), is highly prevalent on admission and is associated with both an excess of in-hospital mortality and an increase in the length of hospital stay (3, 4, 5).

More than 150 million people have been infected by severe acute respiratory syndrome coronavirus-2 (SARS-CoV-2), rendering the coronavirus disease-2019 (COVID-19) pandemic the greatest global public health crisis of this generation. A significant proportion of patients with COVID-19 are hospitalised with viral pneumonia which can progress to severe disease, characterised by various types of organ dysfunction, including septic shock, acute respiratory distress syndrome (ARDS), acute kidney injury (AKI), acute cardiac injury, neurological complications (6), and disseminated intravascular coagulation (DIC) (7). A significant association of adverse outcomes in patients with COVID-19 with several laboratory abnormalities has been shown, including lymphocytopenia, anaemia, thrombocytopenia, hypoalbuminaemia, increased neutrophil count, lactic dehydrogenase (LDH), C-reactive protein (CRP), alanine aminotransferase (ALT), aspartate aminotransferase (AST), urea, creatinine, creatine kinase (CK), erythrocyte sedimentation rate (ESR), ferritin, troponin I, D-dimer, interleukin 6 (IL-6), and interleukin 10 (IL-10) levels (8, 9, 10, 11, 12, 13). In addition, fasting blood glucose concentration on admission above 126 mg/dL (7 mmol/L) (14) and a serum cortisol concentration above 27 mcg/dL (744 nmol/L) (15) are independent predictors for mortality in patients with COVID-19. Regarding the prevalence and prognostic impact of dysnatraemia (abnormal serum sodium levels) in patients with COVID-19, some important publications have appeared in this area since September 2020. In this review, we summarise the literature to date with regards to the prevalence, aetiology, and prognostic value of alterations in serum sodium levels in hospitalised patients with COVID-19. We also provide an overview of the potential pathophysiological mechanisms of hyponatraemia, underpinning the association between sodium values and the magnitude of the inflammatory response, supplemented by recommendations for the optimal investigation and management of hyponatraemia in this context.

## Methods

We undertook a search via *PubMed* and the Cochrane Library until 16th March 2021 of all studies which included the key words 'COVID-19', 'SARS-CoV-2', 'hyponatraemia', 'hypernatraemia', 'sodium', and 'syndrome of inappropriate antidiuretic hormone secretion (SIADH)'. However, of the numerous studies flagged up, only six were found to have examined the prevalence of sodium abnormalities, and their association with clinical outcomes in COVID-19 patients (Table 1), which are reviewed in detail below.

**Table 1:** Prevalence of sodium abnormalities on admission in main observational studies.

Study	COVID-19 patients,* n*	Location	Sodium levels (nmol/L) on admission		
			<135^*^ (%)	<130 (%)	>145^†^ (%)
Frontera *et al.* (1)	4645	4 hospitals, New York	30	7.3	4.1
Hu *et al.* (2)	1254	3 hospitals, China	9.9	NR	2.4
Tezcan *et al.* (3)	408	1 hospital, Turkey	35.8	NR	0
HOPE-COVID-19 (4)	4664	37 hospitals, 7 countries^‡^	20.5	3.8	3.7
Atila *et al.* (5)	172	1 hospital, Switzerland	29.1	NR	2.9
Tzoulis *et al.* (6)	488	2 hospitals, London	24.6	6.2	5.3

*Hyponatraemia; ^†^Hypernatraemia; ^‡^Spain (81%) and Italy (10%).

NR, not reported.

### Prevalence, risk factors, and aetiology of dysnatraemia in COVID-19

The emerging data from six recent observational studies (16, 17, 18, 19, 20, 21) confirm that, in line with other respiratory infections, alterations of serum sodium levels, mainly hyponatraemia, are common in COVID-19 (Table 1). A study, including 4645 hospitalised patients with confirmed SARS-CoV-2 admitted to four New York City hospitals, showed a 30% prevalence of hyponatraemia on admission (16). Older age and lower BMI were independent risk factors for hyponatraemia, but only a small cohort of 36 patients with a serum sodium ≤ 120 mmol/L had data regarding its aetiology (16). In this subgroup, hypovolaemic hyponatraemia and SIADH were equally prevalent, affecting 36% of cases each, in addition, 22% were classified as euvolaemic hyponatraemia owing to low solute intake and 6% as hypervolaemic hyponatraemia (16). Another study of 1254 patients in China showed a 9.9% prevalence of hyponatraemia on admission, although without stating the aetiology of hyponatraemia (17). Hyponatraemia was associated with older age, more co-morbidities, more severe radiological lung findings, and higher levels of neutrophils and CRP (17). Analysis of the international HOPE-COVID-19 registry (Health Outcome Predictive Evaluation for COVID-19), including 4664 hospitalised adults with pneumonia and a positive reverse-transcriptase PCR (RT-PCR) for SARS-CoV-2 (19), reported a prevalence for mild/moderate to severe hyponatraemia (serum sodium 130–134 mmol/L and <130 mmol/L) of 16.7 and 3.8%, respectively (19). Age ≥ 70 years, male gender, chronic kidney disease, tachypnoea, and bilateral pneumonia were risk factors for hyponatraemia (19). An observational cohort study, comparing the prevalence of dysnatraemia and its prognostic impact between cases of COVD-19 and a control group of patients with suspected COVID-19 and similar symptoms, but negative SARS-CoV-2 PCR testing, showed that patients with COVID-19 had a two-fold higher rate of hyponatraemia on admission (20). The only longitudinal study of sodium abnormalities in patients with COVID-19 has reported that almost two-thirds of inpatients experienced dysnatraemia, with 36.9% of patients developing hyponatremia, 10.9% hypernatraemia, and 14.3% both hypernatremia and hyponatremia (21). Using a plasma urea concentration of 5mmol/L as the cut-off value to differentiate euvo- from hypovolaemic hyponatraemia, 56% of cases were classified as probable hypovolaemic and 44% as non-hypovolemic hyponatremia (21). However, there are only limited data regarding the different causes of hyponatraemia, suggesting that SIADH and hypovolaemic hyponatraemia are the commonest causes in COVID-19 (16, 21).

### Association of sodium with disease severity, need for ventilation, and mortality

Many studies have demonstrated the link between serum sodium values on admission with the likelihood of progression to severe illness. Three meta-analyses have reported that patients who develop severe or critical COVID-19 have significantly lower serum sodium concentrations compared to those who do not develop severe COVID-19 (12, 13, 22). These studies indicated a significant association between severe COVID-19 and lower serum sodium concentrations on admission, but the clinical relevance of these small differences of sodium levels, in the range of 0.91–1.97 mmol/L, between patients with severe and non-severe illness, remains questionable.

Table 2 summarises the key findings regarding the association of hyponatraemia with key clinical outcomes in COVID-19 from the six main observational studies (16, 17, 18, 19, 20, 21). It appears that hyponatraemia is associated with a greater need for any form of respiratory support (17, 21), increased likelihood of invasive mechanical ventilation (IMV) with an odds ratio (OR) from 1.83 to 3.30 (16, 18, 20), higher ICU admission rates (OR 2.80–3.73) (18, 20), and the more frequent development of severe sepsis (19) compared to normonatraemic patients.

**Table 2:** Association of hyponatraemia on admission with ICU admission, need for mechanical ventilation, and mortality rate in main observational studies.

Study	ICU admission rate		Applies to	Need for IMV			Mortality		
	OR (95% CI)	*P*		Cases vs normal Na	OR (95% CI)	*P*	Cases vs normal Na	OR (95% CI)	*P*
Frontera *et al.* (1)	N/A		Na <130 mmol/L	46.6% vs 19.4%	1.83 (1.50–2.25	<0.001	26.6% vs 13.2%	1.43 (1.08–1.88)	0.012
Hu *et al.* (2)	N/A		Any form of respiratory support^‡^	87.1% vs 60.1%		<0.001	16.1% vs 6.3%		<0.001
Tezcan* et al.* (3)	3.73 (1.93–7.21)	<0.001			3.20 (1.47– 6.99)	0.003		10.33 (1.62–65.62)	0.01
HOPE-COVID-19 (4)			Composite endpoint of ICU, IMV		1.35 (1.02–1.78)	0.035		1.73 (1.28–2.34)	<0.001
Atila* et al.* (5)	2.80 (1.64–4.88)	<0.001			3.30 (1.67– 6.63)^*^	<0.001		1.40 (1.10–16.62)^†^	0.05
Tzoulis* et al.* (6)	N/A		NIV or IMV		2.18 (1.34– 3.46)	0.0011			NS

^‡^Oxygen, NIV, IMV; ^*^MV only; ^†^Value is HR (95% CI).

ICU, intensive care unit; IMV, invasive mechanical ventilation; NIV, non-invasive ventilation; N/A, not applicable; NS, no significant association.

Several studies have also identified hyponatraemia on admission as an independent risk factor for mortality, with ORs of 1.40 (20), 1.43 (16), 1.73 (19), and 10.33 (18). Despite the higher mortality rate in hypovolaemic or hypervolaemic hyponatraemia compared to that related to SIADH in general hospital populations (23), most studies in the context of COVID-19 have not classified hyponatraemia into subtypes according to volume status. The only study which differentiated hyponatraemia according to volume status did not find an association for hyponatraemia with excess mortality but reported that an increased mortality rate was found only in patients with hypovolaemic hyponatraemia that was independent of acute kidney injury (21). This raises the question as to whether all types of hyponatraemia are linked with excess mortality, and whether the relative risks differ.

Hypernatraemia is also associated with poor prognosis, such as excess in-hospital mortality, ORs of 2.38 (19), 3.05 (21), and 11.50 (20) and seems to be a worse prognostic factor than hyponatraemia in patients with COVID-19 (20) (21). A plausible explanation could be that hypernatraemia affects disproportionally the oldest and most frail individuals who are at the highest risk of death due to COVID-19.

### Association of hyponatraemia with the length of stay and acute kidney injury

Contrary to the well-established significant association of hyponatraemia with the length of hospitalisation in patients with CAP (4), there have been mixed data regarding the relationship of sodium serum abnormalities with the length of hospital stay in patients with COVID-19. Two studies have found no association between sodium levels and the length of stay in patients with COVID-19 (16, 19), whereas three studies (17, 18, 20) have reported a significantly longer duration of hospitalisation in patients with COVID-19 and baseline hyponatraemia compared to those with normal sodium levels (median length of stay 20 vs 17 days, *P* = 0.022 (17); mean length of stay 8.7 vs 7.2 days, *P* = 0.001 (18). This was probably related to the varying criteria determining the length of hospitalisation in various countries and facilities.

Acute kidney injury is common in hospitalised patients with COVID-19, with a prevalence of around 37% (24). Studies have shown contradictory results about a possible link of hyponatraemia with AKI, with some reporting no association (16, 21), whereas others have documented a higher frequency of acute kidney injury (AKI) in relation to hyponatraemia (17). The distribution of types of hyponatraemia in those with AKI is similar to that in those without AKI (21).

### Risk calculators

As hospitals around the world have been facing an increased influx of patients with COVID-19, there has been an urgent need to develop pragmatic risk stratification tools, incorporating demographic, clinical, radiological, and laboratory parameters, to detect patients at high risk of severe illness and thus optimise resource allocation (25). A critical appraisal of 50 prognostic models showed that the most frequently used prognostic factors are age, gender, number of co-morbidities, imaging features, lymphocyte count, serum CRP, and creatinine, with only two including sodium levels (26). Serum sodium levels are not incorporated in most risk stratification tools and do not currently influence real-life decision-making about the therapeutic strategy in COVID-19 patients.

### Pathophysiology of hyponatraemia

The main aetiology of euvolaemic hyponatraemia in patients with COVID-19 is SIADH via four potential mechanisms. First, increased levels of cytokines, such as IL-6, can directly stimulate the non-osmotic release of arginine vasopressin (AVP) (27, 28). Secondly, the injury to lung tissue and alveolar cells can result in a ventilation-perfusion mismatch and compensatory hypoxic pulmonary vasoconstriction leading to inadequate filling of the left atrium, decreased left atrial stretch, and increased AVP secretion (29). Thirdly, patients with COVID-19 pneumonia, through various stimuli, such as pain, nausea, and medications, can stimulate the direct release of AVP. Fourthly, patients receiving positive pressure ventilation (PPV) can have non-osmotic stimulation of AVP secretion, as pulmonary baroreceptors respond to a reduction in effective arterial blood volume (29).

Euvolaemic hyponatraemia can also be attributed to low solute intake, as reported in 22% of COVID-19 patients with severe hyponatraemia (16). An example is the so-called 'tea and toast' diet, commonly experienced by elderly people who are unable to prepare meals at home and eat simple foods with poor protein and salt content. It is characterised by minimal oral solute intake and subsequent low urinary solute excretion, limiting the nephrons’ capacity for solute-free water excretion and resulting in dilutional hyponatraemia (30). Another cause is the development of the adrenal crisis in patients with secondary or tertiary adrenal insufficiency without adequate glucocorticoid treatment during acute COVID-19 infection (31). Observational data suggest that the normal stress response in hospitalised patients with COVID-19 consists of a marked cortisol release, higher than that observed in individuals with similar symptoms but without COVID-19 (15). In light of the COVID-19-related persistent and significant inflammatory response, patients with a suppressed hypothalamo–pituitary–adrenal axis have a high risk of glucocorticoid deficiency, which may lead to 'inappropriate' AVP release and hyponatraemia (31).

Hypovolaemic hyponatraemia is also commonly observed in COVID-19, characterised by depletion of circulating volume triggering baroreceptor-mediated non-osmotic AVP release (32). The high frequency of volume depletion in COVID-19 illness may be explained by an increase in insensible fluid losses due to pyrexia and tachypnoea, limited oral intake, and possible gastrointestinal manifestations of COVID-19, such as vomiting or diarrhoea (16), the use of diuretics, or overly conservative i.v. fluid administration (33). Finally, patients with COVID-19 may have hypervolaemic hyponatraemia, due to pre-existing conditions such as heart failure, cirrhosis, or nephrotic syndrome.

### Link of sodium with inflammation

The main underlying mechanism involved in the development of hyponatraemia associated with inflammatory conditions is that pro-inflammatory cytokines induce the non-osmotic release of AVP, with IL-6 levels being inversely correlated with the sodium concentration (28, 34). Therefore, hyponatraemia is a good surrogate marker of the degree of inflammatory response and tends to reflect the severity of various infections, including pneumonia, tuberculosis, meningitis, encephalitis, HIV infection, and malaria (35).

A small retrospective study of 29 patients with COVID-19 showed that individuals with high serum IL-6 levels had a significantly lower median serum sodium of 133.1 mmol/L vs 139.6 mmol/L compared to those with normal IL-6 levels, while serum sodium concentration was inversely related to IL-6 levels (36). In addition, administration of tocilizumab, an IL-6 antagonist, to hyponatraemic patients with abnormal IL-6 levels led to a significant 48-h increase in serum sodium from 132.4 mmol/L to 139.6 mmol/L (36). Another larger study showed that the higher the IL-6 levels, the lower the serum sodium concentration, confirming this significant inverse relationship between serum sodium and IL-6 levels (16).

High levels of IL-6, and other cytokines such as IL-1β, IL-10, and interferon γ, in the context of COVID-19 lead to a hyperactive immune response state, called the ‘cytokine release syndrome’ (CRS) or cytokine storm, characterised by elevated cytokine levels in the circulation, acute systemic inflammatory symptoms, and secondary organ dysfunction (37, 38). Potent immunomodulatory medications, such as dexamethasone and IL-6 antagonists, have been effectively used in patients with COVID-19 and severe respiratory complications (39, 40).

Based on the hypothesis that IL-6 receptor blockade may be able to improve patient prognosis through disruption of the cytokine storm, tocilizumab has been evaluated in several studies, with mixed negative (41, 42, 43) and positive results, but overall showing better outcomes and lower mortality rates (44, 45, 46).

## Evaluation and treatment of hyponatraemia in COVID-19

**Figure 1:**
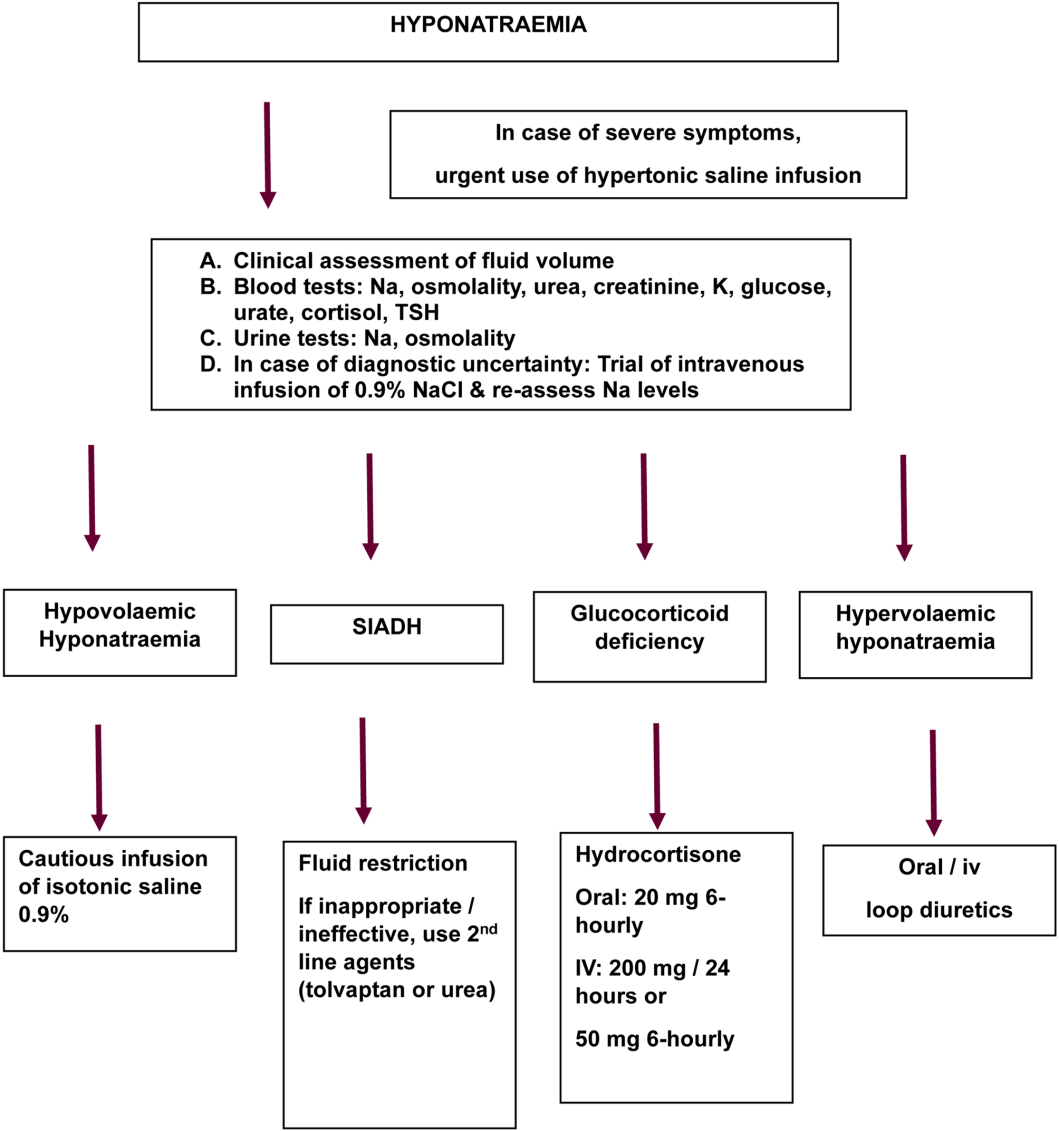
Algorithm for investigation and management of hyponatraemia in patients with COVID-19.

The most appropriate treatment of hyponatraemia necessitates prompt identification of its aetiology, with the therapeutic approach following the principles of managing hyponatraemia in general (Fig. 1) (30, 32). Laboratory evaluation is of paramount importance in view of the low sensitivity and specificity of clinical assessment of volume status in discriminating euvolaemic from hypovolaemic hyponatraemia, the commonest types in the context of COVID-19 (32). Endocrine workup for patients with sodium levels of <130 mmol/L should include measurement of paired serum and urine osmolality and sodium as well as serum glucose, urea, creatinine, urate, cortisol, thyroxine, and thyroid-stimulating hormone (TSH) concentrations (30, 32, 47). Serum urate levels can be useful in distinguishing SIADH from hypovolaemic hyponatraemia, while in cases of diagnostic uncertainty, a trial of volume expansion with i.v. administration of isotonic saline can be a valuable diagnostic and, sometimes in cases of volume depletion, therapeutic tool (30, 47). Although random serum cortisol should be measured to detect new cases of primary or secondary adrenal insufficiency, the serum cortisol concentration cannot be interpreted in patients who are receiving exogenous glucocorticoids, either as part of treatment for acute COVID-19 infection (39) or for pre-existing conditions

Regardless of aetiology, patients with severe symptoms of hyponatraemia, such as seizures, coma, or reduced Glasgow Coma Score, should be promptly treated with hypertonic saline in order to prevent brain herniation, or even, death (30, 32). Evidence and clinical experience are lacking about the optimal form of hypertonic saline treatment in patients with COVID-19. In contrast to contemporary guidelines (30, 32) and recent studies (48) favouring the use of hypertonic saline boluses, continuous infusion of hypertonic saline might be preferable in the context of COVID-19 in order to minimise the risk of pulmonary oedema due to the sudden volume increase (33).

A significant proportion of patients with COVID-19 might not be suitable candidates for fluid restriction, the mainstay of treatment in the general hospital population (30, 32, 47), due to malnutrition, impaired sense of taste, and increased fluid losses due to pyrexia, tachypnoea, and, sometimes, vomiting and diarrhoea (49). In these patients, pharmacotherapy, either tolvaptan or possibly urea, should be considered (30, 47); however, there is very little clinical experience in the use of these agents for the treatment of SIADH patients with COVID-19.

## Future studies

The high prevalence of hyponatraemia and association with poor outcomes in patients with COVID-19 highlight the need for prospective intervention studies in order to determine whether correction of sodium abnormalities might improve clinical outcomes. Specifically, the effectiveness and safety of therapeutic strategies, such as fluid restriction, tolvaptan, and urea, should be evaluated in the context of COVID-19-related SIADH. Studies are needed to explore the pathophysiological basis of hyponatraemia in patients with COVID-19, the frequency of different types of hyponatraemia and their prognostic impact. In order to advance our understanding of the link between inflammatory response and hyponatraemia, prospective studies are warranted to examine longitudinally the serum IL-6 levels, serum sodium concentration, inflammatory markers, and severity of the clinical condition. In addition, the added value of incorporating serum sodium in current risk stratification scores needs to be further explored. These studies will be of value not only in the context of COVID-19 but may also be of value in future pandemics with related viruses.

## Conclusions

Hyponatraemia is highly prevalent at hospital admission in patients with COVID-19 and presents an independent predictor for severe disease, mortality, need for ICU admission, and IMV. In most patients, hyponatraemia is either euvolaemic due to SIADH, mainly owing to IL-6-induced AVP release, or hypovolaemic due to significant insensible fluid loss. Hypernatraemia is also associated with poor clinical outcomes and seems to be an even worse prognostic factor than hyponatraemia.

There is an inverse correlation between sodium and IL-6 levels, suggesting a role for serum sodium as a marker of the inflammatory response. It remains to be seen whether serum sodium should be incorporated in risk stratification scores to enhance their prognostic performance and might also be used for the prompt identification of patients at risk of cytokine storm who may benefit from the early initiation of immunomodulatory agents, such as IL-6 antagonists. It still remains unknown whether correction of hyponatraemia may improve clinical outcomes in patients with COVID-19.

## Declaration of interest

The authors declare that there is no conflict of interest that could be perceived as prejudicing the impartiality of this review.

## Funding

This work did not receive any specific grant from any funding agency in the public, commercial, or not-for-profit sector.
